# *Clostridium botulinum* Type B Isolated From a Wound Botulism Case Due to Injection Drug Use Resembles Other Local Strains Originating From Hawaii

**DOI:** 10.3389/fmicb.2021.678473

**Published:** 2021-07-22

**Authors:** Jessica L. Halpin, Victoria Foltz, Janet K. Dykes, Kevin Chatham-Stephens, Carolina Lúquez

**Affiliations:** Centers for Disease Control and Prevention, Atlanta, GA, United States

**Keywords:** wound botulism, bont/B5, BoNT, *Clostridium botulinum*, heroin use, skin popping, injection drug use

## Abstract

*Clostridium botulinum* produces botulinum neurotoxin (BoNT), which can lead to death if untreated. In the United States, over 90% of wound botulism cases are associated with injection drug use of black tar heroin. We sought to determine the phylogenetic relatedness of *C. botulinum* isolated from an injection drug use wound botulism case and isolates from endogenous infant botulism cases in Hawaii. Nineteen *C. botulinum* type B isolates from Hawaii and one type B isolate from California were analyzed by whole-genome sequencing. The botulinum toxin gene (*bont*) subtype was determined using CLC Genomics Workbench, and the seven-gene multi-locus sequence type (MLST) was identified by querying PubMLST. Mashtree and pairwise average nucleotide identity were used to find nearest neighbors, and Lyve-SET approximated a phylogeny. Eighteen of the isolates harbored the *bont*/B5 gene: of those, 17 were classified as sequence type ST36 and one was classified as ST104. A single isolate from Hawaii harbored *bont*/B1 and was determined to belong to ST110, and the isolate from California harbored *bont*/B1 and belonged to ST30. A tree constructed with Lyve-SET showed a high degree of homology among all the Hawaiian *C. botulinum* isolates that harbor the *bont*/B5 gene. Our results indicate that the *bont*/B-expressing isolates recovered from Hawaii are closely related to each other, suggesting local contamination of the drug paraphernalia or the wound itself with spores rather than contamination of the drug at manufacture or during transport. These findings may assist in identifying interventions to decrease wound botulism among persons who inject drugs.

## Introduction

Botulism is a life-threatening disease caused by botulinum neurotoxins (BoNT) which are produced by *Clostridium botulinum* and rare strains of *Clostridium butyricum* and *Clostridium baratii*. *C. botulinum* is an anaerobic spore-forming bacterium commonly found in soil. Currently, there are seven well-characterized serotypes for BoNT (A–G), classically determined by polyclonal antibody neutralization assays (Hill et al., 2007). Additional serotypes have been proposed (BoNT/X,/En, and/H) but have not yet reached consensus within the scientific community (Barash and Arnon, 2014; Mansfield et al., 2015; Brunt et al., 2018; Popoff, 2018). BoNT are encoded by the *bont* gene, which is part of a cluster with a regulator (*botR*), and non-toxic accessory genes: non-toxic non-hemagglutinin (*ntnh*) and either open reading frame X (*orfX*) or hemagglutinin (*HA*) genes (Rossetto et al., 2014). BoNT serotypes can have multiple gene subtypes, which are determined by differences in the amino acid sequence. Over 40 subtypes of BoNT serotypes A, B, E, and F have been identified to date (Peck et al., 2017). Serotypes A and B cause most human botulism cases within the United States.

Botulinum neurotoxins are metalloproteases that target motor neurons, preventing the release of acetylcholine at neuromuscular junctions, which can lead to a flaccid paralysis (Rossetto et al., 2014). There are four naturally occurring forms of human botulism: infant botulism, foodborne intoxication, wound colonization, and adult intestinal colonization. Infant botulism is the most common form of the disease within the United States. It occurs when a baby under 1 year of age ingests *C. botulinum* spores, most likely by swallowing dust particles that carry locally acquired spores; the spores then germinate within the intestinal tract and produce BoNT *in situ* [Chin et al., 1979;Centers for Disease Control and Prevention (CDC), 1998]. Foodborne botulism occurs when food becomes contaminated with pre-formed BoNT and is ingested [Centers for Disease Control and Prevention (CDC), 1998]. Wound botulism occurs when *C. botulinum* spores enter a wound or necrotic tissue; the spores germinate, multiply, and release BoNT [Centers for Disease Control and Prevention (CDC), 1998]. A very rare form of the disease, adult intestinal colonization, occurs when an adult becomes colonized with *C. botulinum* spores, which germinate and produce BoNT *in situ* [Centers for Disease Control and Prevention (CDC), 1998].

CDC, in partnership with state and local public health laboratories, endeavors to conduct a surveillance for every confirmed case of botulism in the United States. Botulism has been a nationally required notifiable disease since 1947, and a case is confirmed when toxin is detected in clinical samples (e.g., serum, stool) or a suspected food sample or when *C. botulinum* organisms are isolated from a stool sample^1^. CDC’s National Botulism Laboratory serves as a reference laboratory, a central testing laboratory for states that do not do botulism testing, and an overflow laboratory for states who may typically conduct their own botulism testing but require assistance due to capacity or other needs.

The Centers for Disease Control and Preventions’ surveillance data from 1981 to 2016 totals 4,807 laboratory-confirmed cases of botulism within the United States^2^—70% infant botulism, 11% wound botulism, and 19% foodborne or “other” (adult intestinal colonization or unknown route of transmission). Once a rare form of the disease, wound botulism has become more common in the United States starting in the 1990s with the increased use of black tar heroin (BTH) (Passaro et al., 1998; Werner et al., 2000; Davis and King, 2008; Peak et al., 2019). From 1981 to 2016, there was an average of 15 wound botulism cases per year. During this 35-year period, 74.6% of wound botulism cases were due to injection drug use, and 25.4% of wound cases were associated with other types of trauma (e.g., lacerations, abscesses, necrotic tissue, gunshot wounds, or unknown)^2^.

Users of heroin, particularly those who inject subcutaneously (i.e., skin popping), have a higher incidence of wound botulism. Skin popping can create an anaerobic environment under the skin, which facilitates the germination and release of BoNT. Because it is linked to skin popping, black tar heroin is the suspected source of *C. botulinum* spores. Currently, it is unknown exactly how BTH becomes contaminated with *C. botulinum* spores, but there are many opportunities: the drug is often cut with other substances, the drug travels long distances from source to user, the use of dirty needles or equipment, and the drug may be manipulated by the user prior to injection (Davis and King, 2008). Culturing *C. botulinum* from BTH is challenging, as it has a sticky composition that makes it difficult to solubilize in buffers and standard culture media. One of the major challenges for testing BTH is that, because it is a schedule I controlled substance by the United States Drug Enforcement Agency (DEA), a laboratory intending to perform microbiological testing of heroin requires a DEA license and adherence to a strict chain of custody and inventory documentation (United States Department of Justice Drug Enforcement Administration Office of Diversion Control, 2006).

The CDC’s National Botulism Laboratory received isolates from a wound botulism case in Hawaii for confirmation testing. The patient had a history of BTH use, specifically *via* skin-popping daily for 2 weeks prior to hospital admittance. Due to the patient’s history, it is believed that the wound botulism occurred from injection drug use. Based on publicly available CDC surveillance data^2^, this was the first reported laboratory-confirmed case of wound botulism from Hawaii. The isolates were confirmed as *C. botulinum* serotype B. Our analysis of CDC surveillance data for the United States between 1981 and 2016 showed that about 77% (*n* = 420) of reported wound cases in the United States were due to serotype A, 12% (*n* = 65) were attributed to serotype B, and 11% did not have a serotype reported (*n* = 61) (9). Due to the novel report of wound botulism from Hawaii, the isolated geography of the case, and the rarity of serotype B wound botulism, we sought to determine whether the *C. botulinum* type B isolated from this wound botulism case resembled other *C. botulinum* type B isolates from infant botulism cases (approximately 0–4 confirmed cases per year, 1981–2016) in Hawaii (Nevas et al., 2005). *C. botulinum* isolates from infant cases were chosen, as it has been long recognized to be caused by spores from the local environment which become ingested by babies under 1 year old [Centers for Disease Control and Prevention (CDC), 1998]. As a result, these isolates could be considered representative of the local geography in which they occur. A single *C. botulinum* type B isolate from an infant botulism case in California, United States, was included, as it is a common thoroughfare for travel to Hawaii, and it was the geographically closest serotype B environmental isolate found within our collection.

## Materials and Methods

### Microbiology

The strains used in this study are described in Table 1. We recovered each strain from long-term storage by inoculating 0.5–1.0 ml of sporulation media (20 g/L peptone, Difco, Franklin Lakes, NJ, United States; beef brain, Pel-Freez Biologicals; Rogers, AR, United States) into chopped meat glucose starch broth (CMGS) (Remel; Lenexa, KS, United States), and each grew in a Coy (Grass Lake, MI, United States) anaerobic chamber at 35 ± 2°C for 24–48 h. We examined CMGS cultures for growth and quadrant streaked ∼0.5 ml of CMGS culture for isolation onto McClung Toabe egg yolk agar with yeast extract agar plates (McClung Toabe agar base, 75 g/L; yeast extract, 5 g/L; egg yolk enrichment, 100 ml/L—all from Difco, Franklin Lakes, NJ, United States). The plates grew anaerobically at 35 ± 2°C for 24–48 h and were examined for purity. We picked single colonies that exhibited lipase activity and inoculated them into trypticase peptone glucose yeast extract broth (TPGY) (Remel, Lenexa, KS, United States). These cultures grew anaerobically at 35 ± 2°C for 16–24 h prior to genomic DNA extraction.

**TABLE 1:** Summary of *Clostridium botulinum* producing toxin serotype B strains used in this study: year isolated, botulism type, originating state, specimen source, toxin subtype, and seven-gene multi-locus sequence type (MLST).

aIsolate ID #	Year	Botulism type	State	Specimen type	Toxin subtype	Seven-gene MLST
CDC21601	1976	Infant	CA	Stool	B1	30
CDC31747	1986	Infant	HI-Oahu	Stool	B5	36
CDC34293	1989	Infant	HI-Maui	Stool	B5	36
CDC36757	1981	Infant	HI-Oahu	Stool	B5	36
CDC37391	1982	Infant	HI-Oahu	Stool	B5	36
CDC38839	1983	Infant	HI-Oahu	Stool	B5	36
CDC39168	1984	Infant	HI-Maui	Stool	B5	36
CDC40176	1995	Infant	HI-Oahu	Stool	B5	36
CDC41623	1996	Infant	HI-Oahu	Stool	B5	36
CDC45459	1990	Infant	HI-Maui	Stool-enema	B5	36
CDC47455	1992	Infant	HI-Maui	Stool	B5	36
CDC48611	1993	Infant	HI-Kaua’i	Stool	B1	110
CDC49917	1994	Infant	HI-Oahu	Stool	B5	36
CDC53044	2008	Infant	HI-Maui	Stool	B5	36
CDC54117	2009	Infant	HI-Oahu	Stool	B5	36
CDC54250	2009	Infant	HI-Oahu	Stool	B5	36
CDC59947	2004	Infant	HI-Maui	Stool	B5	36
CDC60225	2015	Infant	HI-Hawaii	Stool	B5	36
CDC61035	2016	Wound	HI-Oahu	Wound	B5	36
CDC65069	2010	Infant	HI-Maui	Stool	B5	104

### Genomic DNA Extraction

We used a modified Epicenter (Madison, WI, United States) MasterPure Complete DNA and RNA Purification kit to extract genomic DNA from 8 to 9 ml of turbid TPGY culture. Briefly, cells were pelleted at 4 ± 1°C for 10 min at 4,000 rpm, and the supernatant was discarded. We resuspended cell pellets in lysozyme stock solution (25 mM Tris–HCl, pH 8.0, Invitrogen, Waltham, MA, United States; 2.5 mM 0.5 M EDTA, Invitrogen, Waltham, MA, United States; 10 ml Triton X-100, Sigma, St. Louis, MO, United States; and 20 mg/ml lysozyme from chicken egg white, Sigma, St. Louis, MO, United States) and incubated them for a minimum of 15 min in a 37 ± 1°C water bath. We added 300 μl of undiluted 2X T&C buffer (Epicenter, Madison, WI, United States) and 3 μl of RNase A (Qiagen, Redwood City, CA, United States) to the cell suspension, gently mixed it, and then incubated it in a 57 ± 1°C water bath for 10 min. To this mixture, we added 3 μl of Proteinase K (Invitrogen, Waltham, MA, United States) and incubated it in the water bath for another 10 min at 57 ± 1°C. After incubation with proteinase, we added 350 μl of MPC protein precipitation buffer (Epicenter, Madison, WI, United States) to the solution and centrifuged it at 4,000 rpm at 4 ± 1°C for 10 min. We collected the supernatants and added each to 1 ml of 99% isopropanol to precipitate the DNA. We collected the precipitates, washed them with 1 ml of 70% ethanol, and rehydrated them at least overnight (up to 1 week) in 200 μl of 10 mM Tris–HCl (Invitrogen, Waltham, MA, United States). We filtered the rehydrated gDNA through 0.1-μM centrifugal filters (MilliporeSigma, Burlington, MA, United States) to remove any spores or unlysed cells. We used the Nanodrop 2000 (NanoDrop Technologies, Wilmington, DE, United States) and the Qubit 4 (Invitrogen, Waltham, MA, United States) fluorometer high-sensitivity assay to assess genomic DNA quality and quantity according to the manufacturers’ instructions.

### Whole-Genome Shotgun Sequencing

We constructed barcoded shotgun libraries using NextFlex DNA barcodes (BIOO Scientific, Austin, TX, United States) and the 400-bp Kapa Biosciences (Wilmington, MA, United States) kit for Ion Torrent chemistry (KAPA Biosystems, 2016). We performed size selection using the e-gel system to select for 500-bp fragments (Life Technologies, 2017). We diluted and pooled completed libraries to obtain an equimolar concentration of 200 pM and then templated and enriched using the Ion Chef instrument (Life Technologies), followed by sequencing on Ion Torrent S5 (Life Technologies, 2019).

### Bioinformatics and Quality Control

We assessed read quality using FastQC v.0.11.5 (Andrews, 2014) and assembled reads using SPAdes v.3.10.1 with the following parameters set: sc, iontorrent, single end, and careful (Bankevich et al., 2012). We assessed the resulting assemblies with Quast v.4.3 (Gurevich et al., 2013). We used the map reads to reference tool in CLC Genomic Workbench v.10.1.1 to determine toxin gene subtypes as well as identify accessory genes HA, BotR, and ntnH. We identified the legacy seven-gene multi-locus sequence types (MLST) by querying draft genomes against the PubMLST (Jacobson et al., 2008) database with the Center for Genomic Epidemiology website^3^. We used Mashtree v.0.37 (Katz et al., 2019) to determine pairwise mash distances (Ondov et al., 2016) and place the isolates in a neighbor-joining tree of reference sequences and other serotype B sequences from the CDC short read sequence collection. We used pairwise average nucleotide identity (ANI) (Goris et al., 2007) to identify the closest reference sequences as well as to compare pairwise sequence homologies. We identified the nearest neighbor by ANI, and this reference sequence was used in Lyve-SET v.1.1.4f (Katz et al., 2017) to determine high-quality single-nucleotide polymorphism (hqSNP) sites across the study sequences and then draw a tree with bootstrap support to approximate a phylogeny (Lyve-SET settings enabled: single end, allowed Flanking 5, min_alt_frac 0.75, min_coverage 10, mask-phages, mask-cliffs, read_cleaner = CGP, mapper = smalt). Using these settings, Lyve set defines high-quality SNPs as those in regions of contiguous coverage of at least 10, present in at least 75% of the reads, and not in known phage sequences.

### Data Availability

The Short Read Archive accession numbers are as follows: SHBZ00000000, SHCA00000000, SHCB00000000, SHCC00000000, SHCD00000000, SHCE00000000, SHCF00000000, SHCG00000000, SHCH00000000, SHCI00000000, SHCJ00000000, SHCK00000000, SHCL00000000, SHCM00000000, SHCN00000000, SHCO00000000, SHCP00000000, SHCQ00000000, SHCR00000000, and SHCS00000000.

## Results

The resulting number of reads, GC content, and estimated genome size are found in Table 2. The number of resulting reads was 227,559 to 2,461,814; GC content ranged from 27.7 to 28.0%; reads assembled into 35–257 contigs; N50s ranged from 26,682 to 277,264; average coverage ranged from 20.4× to 227.7×; and approximate assembled genome size was 3.9 to 4.2 mega-bases. Subtypes of Hawaii *C. botulinum* infant or wound isolates were determined to be *bont*/B5 (*n* = 18) and *bont*/B1 (*n* = 1), with an average coverage across the gene of 23× (3–51×). Querying the PubMLST database, which generates an allelic profile or seven-gene MLST sequence type (ST), revealed that the strains harboring *bont*/B5 gene were all members of ST-36 except one, which was ST-104. The *C. botulinum* isolate from Hawaii harboring *bont*/B1 gene was a member of ST-110, and the *C. botulinum* isolate from California harboring a *bont*/B1 gene was a member of ST-30 (Table 1).

**TABLE 2:** Summary of the sequencing statistics for *Clostridium botulinum* strains isolated from infant and wound botulism cases in Hawaii and California.

Isolate ID #	GC (%)	# contigs	Largest contig (bp)	Total length (bp)	N50 (bp)	# reads	Average read length (bp)	Average coverage	# reads mapping to *bont*	Average coverage across *bont*
CDC21601	28.0	55	686,068	3,978,905	277,264	1,916,257	284	143.2	965	69.31
CDC31747	27.7	64	594,898	4,15,0368	224,881	2,461,814	293	180.3	649	44.26
CDC34293	27.7	85	594,976	4,227,167	118,018	1,593,628	293	116.7	333	23.02
CDC36757	27.7	35	653,290	4,156,210	255,842	2,567,944	337	227.7	642	50.47
CDC37391	27.7	79	278,989	4,139,909	128,587	522,334	333	45.8	120	9.36
CDC38839	27.7	41	501,509	4,158,619	301,466	849,846	307	68.7	209	15.24
CDC39168	27.8	193	177,384	4,141,306	46,127	895,235	282	63.1	158	10.25
CDC40176	27.7	116	416,804	4,158,067	75,335	626,019	207	32.4	61	3.24
CDC41623	27.7	59	540,802	4,217,736	194,214	1,283,745	227	72.9	368	20.62
CDC45459	27.7	87	322,199	4,198,117	111,949	2,068,237	254	131.3	498	29.75
CDC47455	27.7	80	273,668	4,199,837	114,010	1,361,983	262	89.2	310	19.07
CDC48611	28.0	122	253,643	3,843,066	53,585	624,594	274	42.8	286	18.95
CDC49917	27.7	252	104,953	4,163,377	26,682	757,736	200	37.9	888	44.96
CDC53044	27.8	86	587,411	4,331,526	225,901	1,788,944	308	137.7	452	33.91
CDC54117	27.7	97	213,589	4,203,614	90,518	648,044	232	37.6	172	9.66
CDC54250	27.7	257	148,760	4,162,820	32,556	227,559	340	20.4	43	3.51
CDC59947	27.7	131	215,257	4,187,706	65,567	615,768	273	42.0	181	12.55
CDC60225	27.7	68	439,777	4,333,055	142,003	1,031,444	334	90.7	254	12.63
CDC61035	27.7	78	430,766	4,153,245	133,216	845,541	288	60.9	209	13.69
CDC65069	27.7	41	636,062	4,186,206	255,806	896,025	334	78.8	227	15.53

**FIGURE 1:**
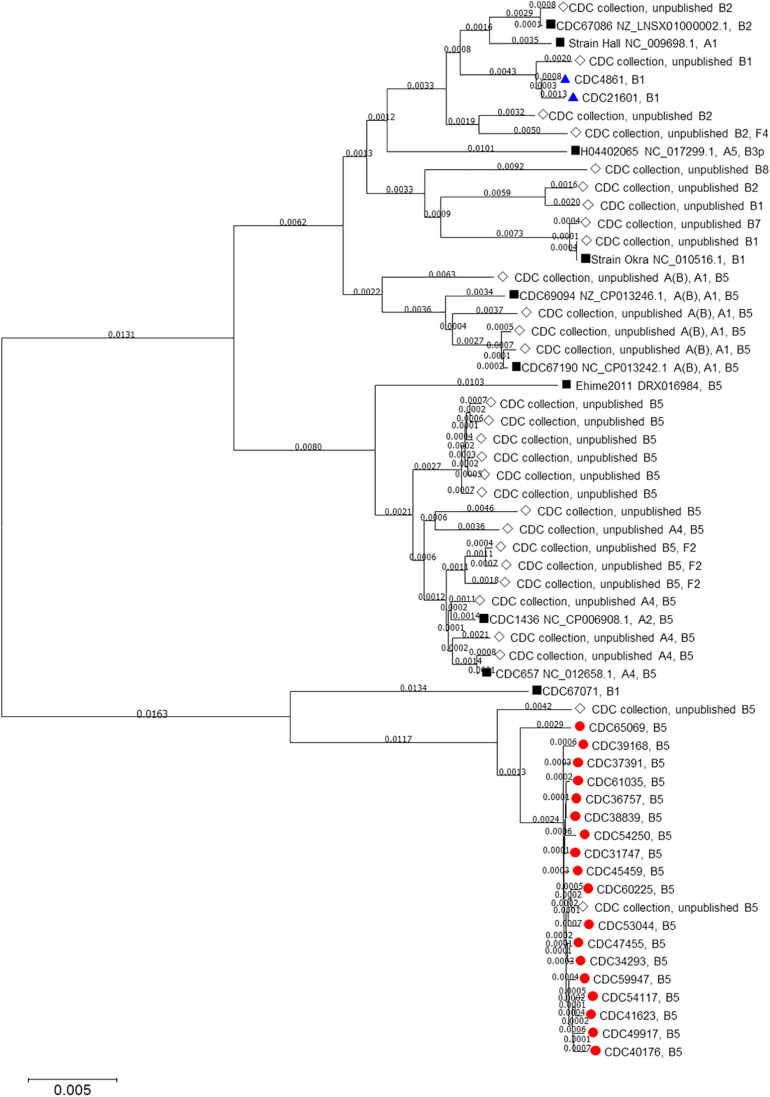
Neighbor-joining tree drawn using MASH distances. The tree includes *Clostridium botulinum* isolates harboring *bont*/B5 (red circles) and *bont*/B1 (blue triangles) genes as described in Table 1 as well as NCBI reference sequences (black squares) and other unpublished isolates from the CDC collection (white diamonds).

Mashtree placed the *C. botulinum* bont/B5 isolates from Hawaii into a single cluster, distant from bivalent strains and other strains that harbor the *bont*/B5 gene (Figure 1). The sole California isolate as well as the isolate from Hawaii harboring *bont*/B1 were members of the *bont*/B1 cluster. ANI corroborated the mashtree results by identifying CDC67071 (RefSeq accession numbers NZ_CP013242.1 and NZ_CP013241.1) as the closest closed reference strain to the Hawaii cluster and verifying that the *C. botulinum* type B isolates from Hawaii are more closely related to each other than to other isolates tested by pairwise ANI.

**FIGURE 2:**
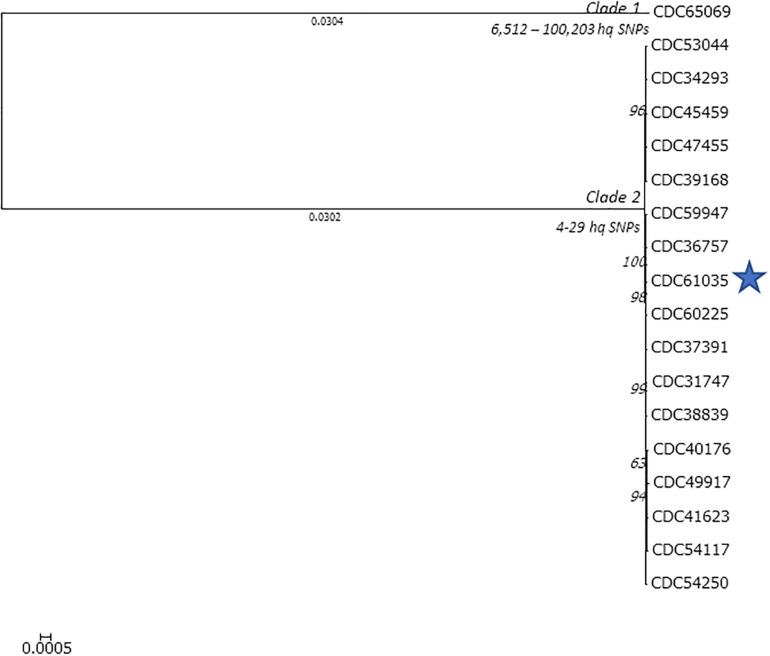
Lyve-SET phylogenetic tree of the *Clostridium botulinum* isolates harboring *bont*/B5 from Hawaii. The blue star represents the isolate from the wound botulism case. Branch lengths < 0.0001 are hidden, and bootstrap values are shown in italics. Lyve-SET was run with the following settings enabled: single end, allowed flanking = 5, min alt frac = 0.75, minimum coverage = 10, mask-phages, mask-cliffs, read cleaner = CGP, mapper = smalt. The reference used to identify high-quality single-nucleotide polymorphism sites was CDC67071 (NZ_CP013242.1).

We used Lyve-SET with CDC67071 as the reference to approximate a phylogeny of the *C. botulinum* isolates from Hawaii that harbor *bont*/B5 (Figure 2). Lyve-SET used 10,424 hqSNP sites to approximate a phylogeny which resulted in 4–10,103 pairwise hqSNP differences between the sequences. Within this tree, two clades formed: clade 1 containing CDC65069 (ST-104, harboring bont/B5 gene) from an infant botulism case and clade 2 containing CDC31747, CDC34293, CDC36757, CDC37391, CDC38839, CDC38168, CDC40176, CDC41623, CDC45459, CDC47455, CDC49917, CDC53044, CDC54117, CDC54250, CDC59947, CDC60225, and CDC61035 (wound isolate). Clade 2 contains the remainder of the *bont*/B5 isolates from Hawaii, all belonging to ST36 and all from infant cases except for the single wound botulism isolate (as noted). Clade 1 is separated from clade 2 by 10,103 hqSNP differences, and 4–29 hqSNP differences are found within clade 2.

For these 18 *C. botulinum* type B isolated from botulism cases from Hawaii, the *bont*/B5 gene is located on a plasmid. We found nothing notable about the hemagglutinins or the *bot*R gene, but the *ntnh* gene has a 39-bp insert. This insertion was not in the *ntnh* gene of isolates harboring bont/B1 that we investigated herein nor in reference strains Okra, CDC67071, CDC69094, or CDC1436. This addition does not disrupt the frame of the gene.

## Discussion

The results herein suggest that the heroin that is presumed to be the causative agent of wound botulism was contaminated with *C. botulinum* spores locally rather than in manufacturing or transit. This was the first reported wound case from Hawaii and was confirmed in the laboratory as toxin type B. The CDC surveillance data (1981–2016) reports a total of 61 confirmed cases of botulism from Hawaii (53 infant botulism, seven foodborne botulism, and one wound botulism). Serotype B was confirmed for 56 out of the 61 total cases; the remaining five were infant botulism cases due to serotype A^N1^.

Black tar heroin was first introduced into the United States from Mexico in the 1970s and was predominantly used in California. According to the Hawaii Drug Threat Assessment, the main distribution hub for BTH to Hawaii is through Los Angeles, CA (National Drug Intelligence Center, 2002). Heroin is typically cut where it is distributed; however, it is still unclear if contamination with *C. botulinum* spores occurs from local or distant geographic sources (Passaro et al., 1998; Werner et al., 2000; Peak et al., 2019).

Because of the high number of type B botulism cases in Hawaii, we had the opportunity to select and compare 18 *C. botulinum* type B isolates from the CDC strain collection associated with infant botulism. These isolates were selected for this study to aid in determining if this *C. botulinum* type B isolate from a wound botulism case is phylogenetically related to other locally acquired Hawaiian *C. botulinum* isolates. Infants may acquire *C. botulinum* spores by swallowing dust particles, and thus *C. botulinum* isolated from infant botulism cases can be viewed as environmental representatives of endogenous strains (Nevas et al., 2005; Fleck-Derderian et al., 2017). We also included a California *C. botulinum* type B isolate because the import path for BTH to Hawaii is through California. This isolate is also the only “environmentally acquired” *C. botulinum* type B isolate from the west coast in the CDC historic strain collection.

Eighteen out of the 19 *C. botulinum* type B isolates from botulism cases from Hawaii harbored the *bont/*B5 gene, including the wound isolate (CDC61035). Previous studies investigating *C. botulinum* genetic diversity (Hill et al., 2007; Luquez et al., 2009; Williamson et al., 2016) indicate that a large portion of *bont/*B5-harboring strains has been identified in bivalent strains (types Ab, Ba, and Bf) or type A isolates that harbor a silent B gene [designated as A(B) strains]. These earlier studies indicate that A(B) strains typically cluster together due to their highly conserved sequences, and bivalent strains display more variability within their sequences; however, they still cluster closely. It is known that the *bont*/B5 gene can be found alone (Franciosa et al., 2009; Kenri et al., 2014), and our observation is that the occurrence of *bont*/B5 with no other toxin gene present is more common than once thought (unpublished observations). Williamson et al. (2016) reported that a *C. botulinum* type B strain that harbors only a *bont*/B5 gene still clusters closely to bivalent strains in core genome phylogenies. There have not been extensive studies published studying the evolution of *C. botulinum* strains over time as they persist in the environment.

For these 18 *C. botulinum* type B isolated from botulism cases from Hawaii, the *bont*/B5 gene is located on a plasmid. Each of the contigs containing the *bont* gene was compared against the NCBI nucleotide database using blastn, and the group most closely resembled pCLJ from bivalent strain CDC657, which harbors the *bont*/A4 and *bont*/B5 genes. There was nothing notable about most of the accessory genes (hemagglutanins and BotR), but there is a 39-bp insert in the *ntnh* gene that will not disrupt the reading frame. This insertion is also present in *C. botulinum* strain CDC657 *bont*/B-associated *ntnh* gene (accession #EU341304) but not in the *ntnh* gene of isolates harboring *bont*/B1 that we investigated herein nor in reference strains Okra, CDC67071, CDC69094, or CDC1436. This addition does not disrupt the frame of the gene, allowing a fully functional protein to be produced.

A neighbor-joining tree (Figure 1) of all the isolates used in this study was constructed using their Mash distances along with publicly available *C. botulinum* reference sequences and sequences of other *C. botulinum* type B isolates from the CDC collection. As indicated in Figure 1, **17** isolates harboring *bont*/B5 gene, including the wound isolate (CDC61035), formed their own cluster away from other *bont*/B5 harboring strains. The MLST analysis revealed that these 17 isolates belonged to ST-36. The other isolate harboring *bont/B5* gene, CDC65069, clustered just outside the other 17 isolates and belonged to ST-104. These two sequence types, ST-36 and ST-104, differ at the *recA* and *hsp* loci. These results show that seven-gene MLST and MASH tree clustering provided congruent subtyping results.

One *C. botulinum* type B isolated from an infant botulism case from Hawaii (CDC48611) and the *C. botulinum* type B isolated from an infant botulism case from California (CDC21601) were determined to be subtype *bont/*B1. Both isolates clustered together and away from isolates harboring *bont*/B5 gene. The seven-gene MLST analysis showed CDC48611 as belonging to ST110 and CDC21601 as belonging to ST30. These two sequence types differ by only one locus, at *aceK*. Based on these limited data, it is unclear whether isolates harboring *bont*/B1 are common within Hawaii or whether the Hawaii *bont*/B1 case was due to travel from mainland United States.

LYVE-Set was used to approximate the phylogeny among the 18 *C. botulinum* isolates from Hawaii harboring *bont*/B5 gene (Figure 2). LYVE-Set resulted in two separate clades: one consisting of 17 isolates with 4–29 high -quality SNP differences among them; all these isolates belong to MLST ST-36. The small number of SNPs observed among members of clade 1 indicates a high homology. Clade 2 was separated from clade 1 by 10,103 high-quality SNPs, with only one member, CDC65069, which belongs to ST-104. The LYVE-set results indicate a high homology between the wound isolate (CDC61035) and the other isolates from infant botulism cases, which suggests that the *C. botulinum* type B isolated from a wound botulism case is closely related to other *C. botulinum* isolates present in the environment in Hawaii.

Attempts to identify how BTH becomes contaminated with *C. botulinum* spores have been unsuccessful. Previous studies conducted by the California Department of Public Health suggest that contamination of BTH with *C. botulinum* spores may occur during the cutting and diluting process (Passaro et al., 1998), and a study by Peak et al. suggests that BTH is typically cut where it is distributed (Peak et al., 2019). The Hawaii Drug Threat Assessment indicates that Oahu is the point of entry for Hawaii and is also a major distribution hub. It is also suggested that heroin is cut in Oahu before supplying to sellers and/or users (National Drug Intelligence Center, 2002).

**FIGURE 3:**
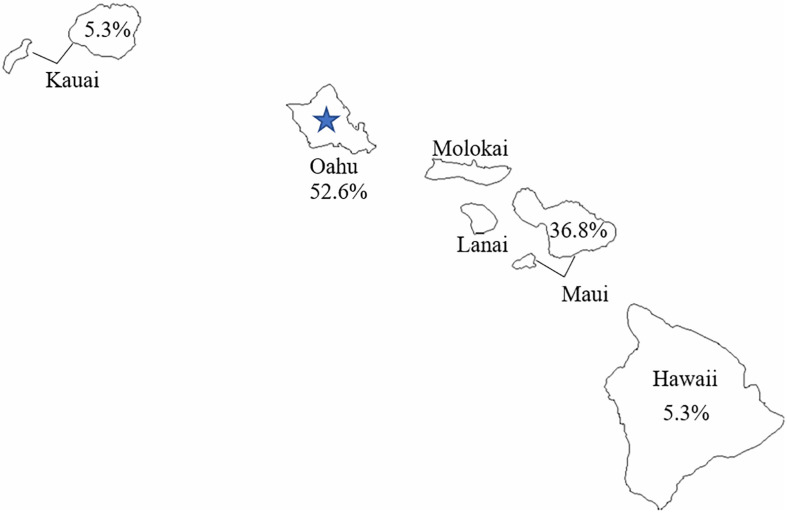
Geographic distribution of *Clostridium botulinum* type B used in this study. The blue star represents the reported origin of the wound botulism case.

The geographic distribution of *C. botulinum* isolates used in this study is relatively broad. Figure 3 shows the percentage of isolates used in the study per Hawaiian island. Oahu had the highest representation of isolates from infant botulism cases used in this study, and it was also the origin of the wound isolate (CDC61035). The close phylogenetic relationship between the *C. botulinum* type B isolated from the wound botulism case and the isolates from local infant botulism cases and the geographic distribution of these isolates in Hawaii strongly supports the hypothesis that this wound botulism case from Hawaii was due to locally acquired *C. botulinum* spores rather than from contamination during BTH production from a distant source.

## Data Availability Statement

The datasets presented in this study can be found in online repositories. The names of the repository/repositories and accession number(s) can be found in the article/Supplementary Material.

## Ethics Statement

The studies involving human participants were reviewed and approved by CDC Institutional Review Board. Written informed consent for participation was not required for this study in accordance with the national legislation and the institutional requirements.

## Author Contributions

JH and CL conceived the study. JH and VF performed the sequencing and data analysis. JD performed the original microbiology and identification of strains. KC-S provided the epidemiological review. All authors contributed to the article and approved the submitted version.

## Conflict of Interest

The authors declare that the research was conducted in the absence of any commercial or financial relationships that could be construed as a potential conflict of interest.

## Footnotes

1 https://wwwn.cdc.gov/nndss/conditions/botulism/case-definition/2011/

2 http://www.cdc.gov/nationalsurveillance/botulism-surveillance.html

3 http://www.genomicepidemiology.org/
