# Supplementary material for: Clostridium botulinum Type B Isolated From a Wound Botulism Case Due to Injection Drug Use Resembles Other Local Strains Originating From Hawaii
Source: Front Microbiol. 2021 Jul 22;12:678473. doi: 10.3389/fmicb.2021.678473 (PMC8339428; doi:10.3389/fmicb.2021.678473)
Supplement: Supplementary file 1 [file Table_1.DOCX]

Supplemental information

*Clostridium botulinum* producing toxin serotype B isolated from a wound botulism case due to injection drug use resembles other local strains originating from Hawaii

Jessica L. Halpin, Victoria Foltz, Janet K. Dykes, Kevin Chatham-Stephens, Carolina Lúquez

| **Isolate ID #** | **HA-70 1881bp** | | **HA-33 882bp** | | **HA-17 441bp** | | **BotR 537bp** | | **ntnH 3630-3594bp** | |
| --- | --- | --- | --- | --- | --- | --- | --- | --- | --- | --- |
|  | # reads mapped | Average coverage across gene | # reads mapped | Average coverage across gene | # reads mapped | Average coverage across gene | # reads mapped | Average coverage across gene | # reads mapped | Average coverage across gene |
| **CDC21601** | 823 | 116.6 | 228 | 62.7 | 89 | 48.4 | 36 | 15.4 | 975 | 74.7 |
| **CDC31747** | 1534 | 230.9 | 425 | 133.8 | 179 | 93.7 | 113 | 48.5 | 1343 | 100.2 |
| **CDC34293** | 822 | 118.0 | 212 | 66.7 | 94 | 50.4 | 66 | 27.0 | 621 | 45 |
| **CDC36757** | 1454 | 234.9 | 336 | 114.7 | 174 | 98.5 | 101 | 46.3 | 1491 | 124.7 |
| **CDC37391** | 268 | 44.2 | 62 | 20.5 | 51 | 15.7 | 19 | 9.1 | 254 | 21.6 |
| **CDC38839** | 443 | 66.6 | 117 | 39.3 | 54 | 30.8 | 37 | 17.1 | 408 | 32.7 |
| **CDC39168** | 336 | 48.1 | 120 | 37.2 | 52 | 25.5 | 15 | 5.4 | 292 | 20.5 |
| **CDC40176** | 100 | 9.9 | 27 | 5.8 | 14 | 6.7 | 8 | 3.3 | 119 | 6.3 |
| **CDC41623** | 666 | 80.3 | 167 | 38.1 | 93 | 45.5 | 61 | 21.9 | 802 | 48.1 |
| **CDC45459** | 1245 | 161.7 | 282 | 77.2 | 111 | 52 | 107 | 39.9 | 1030 | 66.8 |
| **CDC47455** | 747 | 93.5 | 209 | 58.1 | 86 | 39.5 | 53 | 19.4 | 640 | 41.3 |
| **CDC48611** | 302 | 40.6 | 66 | 16.8 | 32 | 15.9 | 22 | 8.8 | 289 | 19.8 |
| **CDC49917** | 1801 | 188.7 | 476 | 102 | 232 | 92.6 | 160 | 52.2 | 1782 | 94.6 |
| **CDC53044** | 812 | 128.3 | 200 | 66.9 | 101 | 58.4 | 73 | 35.6 | 851 | 67.2 |
| **CDC54117** | 344 | 43.8 | 97 | 25.5 | 36 | 17.7 | 42 | 13.4 | 388 | 22.6 |
| **CDC54250** | 107 | 18.8 | 29 | 11.1 | 10 | 6.1 | 5 | 2.5 | 94 | 8.6 |
| **CDC59947** | 332 | 47.3 | 96 | 27.5 | 62 | 29.4 | 33 | 12.7 | 329 | 23.5 |
| **CDC60225** | 339 | 33.7 | 89 | 18.6 | 48 | 18.6 | 39 | 11.8 | 124 | 5.4 |
| **CDC61035** | 514 | 75.3 | 158 | 51.1 | 67 | 34.6 | 41 | 15.9 | 529 | 38.6 |
| **CDC65069** | 640 | 87.4 | 163 | 48.4 | 62 | 32.7 | 47 | 17.9 | 581 | 38.8 |

Table 3. Supplemental information: Table of results for mapping of the accessory genes for Clostridium botulinum type B strains used in this study.
